# Skin and Soft Tissue Infections and Vascular Disease among Drug Users, England

**DOI:** 10.3201/eid1310.061196

**Published:** 2007-10

**Authors:** Charles Irish, Roy Maxwell, Mark Dancox, Paul Brown, Caroline Trotter, Julia Verne, Mary Shaw

**Affiliations:** * Health Protection Agency South West, Bristol, United Kingdom; † University of Bristol Department of Social Medicine, Bristol, United Kingdom; ‡ South West Public Health Observatory, Bristol, United Kingdom

**Keywords:** intravenous drug abuse, groin, femoral vein, abscess, cellulitis, thrombophlebitis, pseudoaneurysm, letter

**To the Editor:** The injecting of illicit drugs is associated with skin and soft tissue infections (SSTIs) and vascular disease (*1*–*3*). These conditions include the development of cutaneous abscess and cellulitis at injection sites, from subcutaneous or intramuscular injecting, known as skin and muscle popping, and intravenous injecting (*1*–*3*). Intravenous injection is associated with phlebitis or thrombophlebitis, in which the vein may become infected (*4*). Inadvertent arterial injection, particularly when attempting to inject into the femoral vein, that is, “groin injecting,” may cause arterial pseudoaneurysm (*4*,*5*).

Drug-related conditions form a major part of the workload of some hospital emergency departments in the United Kingdom and elsewhere (*1*–*3*,*6*). We aimed to identify emerging trends in hospital admission for SSTIs and vascular disease arising from drug use and, specifically, where these may have occurred after injection of the femoral vein.

We extracted hospital admission data for drug users 15–44 years of age for the fiscal years April 1, 1997–March 31, 2004, from the UK Department of Health, hospital episode statistics (HES) database. Using the International Classification of Diseases, 10th revision (ICD-10) codes F11–16, F18, and, F19, we identified drug users by a record in any diagnostic field of mental and behavioral disorders due to psychoactive substances, excluding alcohol and tobacco. We identified the primary diagnosis on admission and whether the admission was as an emergency.

Over the study period, admissions of drug users for cutaneous abscess, L020–L029; cellulitis, L030–L039; and phlebitis or thrombophlebitis, I801–I809; increased substantially (Appendix Figure). Increases occurred in specific primary diagnoses from 1997–1998 to 2003–2004; the largest percentage increase was for phlebitis or thrombophlebitis of the femoral vein, I801, from 60 to 533 (788%). Increases were also observed for aneurysm or pseudoaneurysm of an artery of a lower limb, I724, from 9 to 62 (589%), cutaneous abscess of trunk or groin, L022, from 92 to 613 (566%), cellulitis of trunk or groin, L033, from 13 to 74 (469%), and, phlebitis or thrombophlebitis of deep vessels of the lower limb other than the femoral vein, I802, from 269 to 1,314 (388%).

No national data exist for the prevalence of injection drug use in England (*7*). Although the number of opiate injecting drug users may have increased in the 1990s (*8*), the rapid and substantial increase in admissions for SSTIs and vascular disease suggests that this has not resulted from an increase in the injecting population alone.

The contribution to the increase in admissions from subcutaneous or muscle injecting and intravenous injecting cannot be determined from these data. The increases for phlebitis or thrombophlebitis of the femoral vein and aneurysm or pseudoaneurysm of the lower limb suggest that groin injecting may have contributed to the study findings (*9*). The choice of drugs may have contributed to our findings. An association between injecting site infections in England has been reported with crack cocaine injection and elsewhere with cocaine injection (*2*,*3*,*7*).

This study has some limitations. The HES database does not distinguish between injection and noninjection drug users and whether injection was intravenous or subcutaneous and intramuscular. The study does not relate HES data entries to the conditions described directly by the physical examination of patients or the review of clinical notes. Those conditions associated with femoral vein injection do not exclusively result from this practice, and the proportion of these admissions not associated with injection drug use is unknown. The study period was limited by date of the introduction of ICD-10 coding; therefore, earlier trends could not be identified. Nevertheless, this analysis highlights a potentially important trend and the need for further quantitative and qualitative research in injection drug users.

The response to these problems could be addressed by changing behavior and improving access to healthcare (*1*). Ideally, injection drug users (IDUs) should have early entry to, and be retained on, substance abuse treatment, particularly methadone maintenance (*1*). Skin and muscle injecting, and injecting into the femoral vein should be discouraged (*3*). To inject safely, IDUs need access to clean equipment to prevent the use of shared and dirty needles and the reuse of syringes. Injection sites should be rotated, the skin should be cleaned with alcohol, and the licking of needles and booting should be discouraged (*1*–*3*). Patients were predominantly admitted through emergency departments, which suggests poor contact with health services and reluctance to seek treatment until the point of crisis (*10*). Early medical treatment is required, possibly with the creation of hospital-based SSTI clinics, as were successfully introduced in San Francisco, or improved community outreach (*1*).

In summary, this study identifies a rapid and important increase in the hospitalization of drug users in England for SSTIs and vascular conditions. Further work is required to obtain more information about these clinical problems and the patients’ associated lifestyle, on admission and in the community. Means of discouraging risk-related behavior and treatment should be implemented before the conditions require urgent hospital admission.

## Supplementary Material

Appendix Figure Hospital admissions of drug users in England for phlebitis or thrombophlebitis, cellulitis, or cutaneous abscess as a primary cause, for the fiscal years 1997-1998 to 2003-2004. Source: UK Department of Health hospital episode statistics.
