# Supplementary figures and images for: Skin and Soft Tissue Infections and Vascular Disease among Drug Users, England
Source: Emerg Infect Dis. 2007 Oct;13(10):1510–1. doi: 10.3201/eid1310.061196 (PMC2851502; doi:10.3201/eid1310.061196)

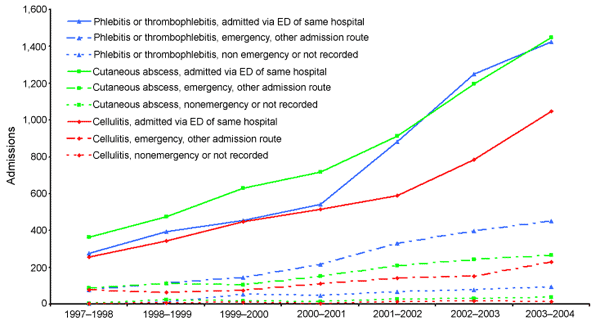

Supplement: Appendix Figure — Hospital admissions of drug users in England for phlebitis or thrombophlebitis, cellulitis, or cutaneous abscess as a primary cause, for the fiscal years 1997-1998 to 2003-2004. Source: UK Department of Health hospital episode statistics. [file 06-1196_appF-s1.gif]
